# Do Osmolytes Impact the Structure and Dynamics of Myoglobin?

**DOI:** 10.3390/molecules23123189

**Published:** 2018-12-03

**Authors:** Dorota Kossowska, Kyungwon Kwak, Minhaeng Cho

**Affiliations:** 1 Center for Molecular Spectroscopy and Dynamics, Institute for Basic Science (IBS), Seoul 02841, Korea; kossowska.dorota.ewa@gmail.com; 2 Department of Chemistry, Korea University, Seoul 136-713, Korea

**Keywords:** IR spectroscopy, IR pump-probe, vibrational spectroscopy, ultrafast vibrational dynamics, vibrational probe, IR probe, myoglobin, protein dynamics, myoglobin, osmolytes

## Abstract

Osmolytes are small organic compounds that can affect the stability of proteins in living cells. The mechanism of osmolytes’ protective effects on protein structure and dynamics has not been fully explained, but in general, two possibilities have been suggested and examined: a direct interaction of osmolytes with proteins (water replacement hypothesis), and an indirect interaction (vitrification hypothesis). Here, to investigate these two possible mechanisms, we studied myoglobin-osmolyte systems using FTIR, UV-vis, CD, and femtosecond IR pump-probe spectroscopy. Interestingly, noticeable changes are observed in both the lifetime of the CO stretch of CO-bound myoglobin and the spectra of UV-vis, CD, and FTIR upon addition of the osmolytes. In addition, the temperature-dependent CD studies reveal that the protein’s thermal stability depends on molecular structure, hydrogen-bonding ability, and size of osmolytes. We anticipate that the present experimental results provide important clues about the complicated and intricate mechanism of osmolyte effects on protein structure and dynamics in a crowded cellular environment.

## 1. Introduction

A detailed knowledge of protein structure and dynamics and the relation between them is essential for a thorough understanding of protein function. A variety of analytical techniques such as X-ray diffraction [1,2], NMR [3,4], 2D-NMR [5], and neutron diffraction [6] have been used in order to study the structures of proteins, nucleic acids, and various small biomolecules.

Protein dynamics occur on time scales covering a range from sub-picoseconds to microseconds or even longer. All of the aforementioned techniques have proven to be powerful tools to investigate protein structure, but they are of limited use in identifying and monitoring rapidly interconverting conformational substrates due to their low-time resolutions. Thus, protein dynamics have been investigated by means of time-resolved spectroscopic methods like fluorescence [7], IR pump-probe [8,9], two-dimensional IR spectroscopy (2D IR) [10,11,12], vis/vis and vis/mid-IR pump-probe [13,14], pump-probe-type X-ray solution scattering, etc. [15,16], However, even spectroscopic techniques with sufficiently high time resolution, which are capable of probing ultrafast dynamics, still cannot provide atomic scale information on structural dynamics. Thus, computational techniques such as molecular dynamics (MD) simulations and quantum chemistry calculations have been found to be of great help in interpreting various spectroscopic observations to ultimately obtain a molecular-level understanding of protein motions and functions occurring on sub-nanosecond time scales [17,18,19].

Myoglobin (Mb), a small globular protein consisting of 153 amino acids with a mass of 18 kD [20] carrying oxygen in muscles, has long served as a model system for both experimental and computational studies of protein dynamics and its relation to function and structure. The presence of the protoheme, a chromophoric prosthetic group also denoted as protoporphyrin-IX, in the Mb structure allows it to reversibly bind small ligand such as O_2_, CO, or NO [21]. Upon ligand binding, the conformation of Mb changes, as shown in X-ray diffraction studies. Structures of the ligated carbonmonoxy myoglobin (COMb) and de-ligated Mb, as well as binding kinetics of CO at the active site of Mb, have been investigated using a variety of techniques like X-ray crystallography [22], Raman spectroscopy [23], circular dichroism [24,25], UV-vis and time-resolved optical spectroscopy [26,27], ^13^C-NMR [28,29], and FTIR and time-resolved IR spectroscopy [30,31]. COMb is an excellent model system for such time-resolved spectroscopic studies because of its high chemical stability in solution, almost unitary quantum efficiency for photolysis, and ultrafast (<50 fs) photolyzability by visible pulses [32].

Both FTIR and 2D IR spectroscopies have been found to be of exceptional use in characterizing Mb endogenous amide bands [33] and exogenous ligands such as CO, NO, CN, SCN, SeCN, etc. [11,34,35,36,37] One of the most extensively-used IR active ligand binding to the Mb heme is CO. Its FTIR band appears in the transparent window of the protein IR spectra. Furthermore, due to its high dipole strength, a relatively small amount of protein sample is needed for both IR pump-probe and 2D IR studies. Additionally, the relatively small size of COMb allows researchers to perform MD simulations over the time scales long enough to study both equilibrium and non-equilibrium behaviors, which could be used to directly compare with experimental results [38,39]. 

In the present work, we study osmolyte effects on the structure of COMb. Generally, living cells need to counter-balance any deleterious external or internal perturbations at the physiological conditions to maintain the secondary and tertiary structures of proteins [40,41]. One of the most important cellular processes in response to external stresses is to accumulate small organic molecules called osmolytes (or osmoprotectants) in live cells [42,43]. Osmolytes are defined as compounds affecting osmosis, and this definition covers variety of different compounds groups such as inorganic salts, organic osmolytes—polyols, sugars, amino acids and others. The concentration of the osmolytes in cells can vary depending on the type of the organism, type of the cell itself, and the current condition of the cell environment. There is not a lot of numerical data published for osmolytes concentrations in the physiological systems, but the osmolarity of the osmolytes in the mammalian blood is reported to be ~290 mOsmol/kg [44], and in canine kidney cells it can reach even ~600 mOsmol/kg [45]. Often, certain osmolytes play an important role in maintaining cell volume and fluid balance. They sometimes have a direct impact on protein stability and solubility [46,47], contribute to protein folding [42,48], or modulate the tendency of proteins to aggregate and the nature of aggregates [49]. Numerous papers considering the effects of osmolytes on protein structure have been published, but the majority of them focus on trehalose [50,51,52]. Preferential interaction, thermal unfolding, and thermotolerance studies show that trehalose stabilizes proteins in their native state at high temperatures in living cells, and that it reduces the aggregation of denatured proteins, and stabilizes the proteins most probably by general weak protein-co-solvent interactions [50,52]. The results of the MD simulations point to a water entrapment scenario of the protein stabilization by trehalose, in which very slow water is trapped between the protein and a layer of trehalose molecules [51]. Few papers about sorbitol [53] and other osmolytes have been published, but the studies with osmolytes different than trehalose often focus on protein stabilization in food samples [54]. However, the exact mechanism explaining how these osmolytes stabilize proteins is still unclear, despite the fact that it has been the subject of extensive research. Two main hypotheses have been considered: (i) the water replacement hypothesis [55,56,57], which is based on the assumption that osmolytes directly interact with proteins by replacing hydration water on the surface of proteins with osmolyte molecules, and (ii) the vitrification hypothesis [58], which assumes that osmolytes indirectly affect protein structure and function. In the latter case, the osmolyte molecules interact with solvent molecules (water), which results in a change in protein solvation. Here, we specifically investigate the influences of sorbitol, glycine betaine, trehalose, taurine, and myo-inositol on the structure and dynamics of myoglobin and carbonmonoxy myoglobin by employing UV-vis absorption, circular dichroism, FTIR, and time-resolved IR pump-probe measurement methods.

## 2. Results and Discussion

### 2.1. UV-vis Spectroscopy

**Figure 1:**
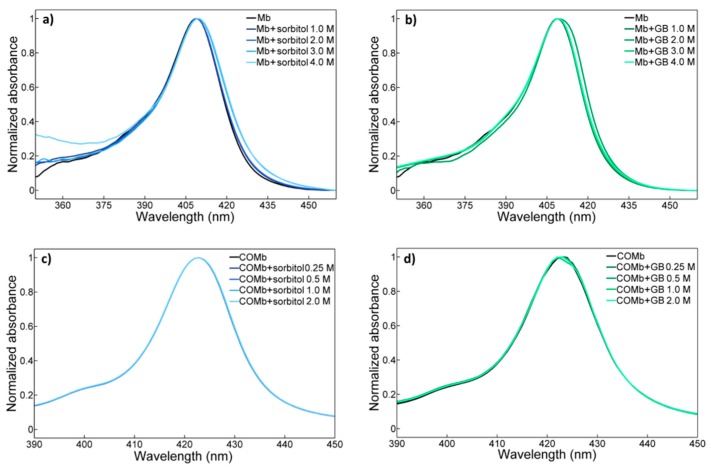
Concentration dependent UV-vis spectra of (**a**) metmyoglobin in the sorbitol solutions, (**b**) metmyoglobin in the glycine betaine solutions, (**c**) carbonmonoxy-myoglobin in the sorbitol solutions, and (**d**) carbonmonoxy-myoglobin in the glycine betaine solutions.

We first performed a measurement of the Soret (Figure 1) and Q bands (Figure S1) of both Mb and COMb in osmolyte (sorbitol and glycine betaine) solutions. The lineshapes and peak positions of the two bands of Mb are found to be in agreement with those in the literature [27,59]. In the case of Mb, we observe an asymmetric Soret band with a maximum at around 410 nm and a wide shoulder band. The Q bands peak at 510 nm and its lineshape is also asymmetric. Upon binding of CO to the oxidized Mb, the Soret band of COMb is red-shifted with peak maximum at 420 nm and its lineshape changes toward a more symmetric Gaussian shape with a weak shoulder band at around 400 nm. The more pronounced change can be found in the Q band region upon the ligation of CO into Mb. Instead of one asymmetric peak, the Q band appears to be a doublet at 540 nm and 577 nm. Such changes in the UV-vis spectra of COMb, as compared to those of Mb, show that the oxidation of Mb, and CO purging, resulted in a successful preparation of COMb. 

Next, a series of UV-vis spectroscopic studies of both myoglobin and carbonmonoxy myoglobin in solutions of different osmolytes with varying concentrations was performed. The Soret bands of Mb and COMb in sorbitol and glycine betaine solutions are shown in Figure 1. The addition of these osmolytes induces a peak shift and a change in their lineshapes. However, those changes induced by dissolved osmolytes at the concentrations used (≤ 4 M) are marginal, and it is difficult to observe any dependence of the UV-vis spectrum on osmolyte concentration. The same is true of carbonmonoxy myoglobin. The Soret band lineshape of COMb in sorbitol or glycine betaine solutions is almost identical to that of COMb in water. The UV-vis spectroscopic results for the two proteins in trehalose, taurine, and myo-inositol solutions show the same pattern (see Figure S3). 

In the myoglobin solution, water molecules not only interact with surface residues of the protein, but also occupy the internal heme pocket [60]. These buried water molecules participate in modulating dynamical processes such as proton transfer processes or protein folding, and usually have a critical influence on the structural stability and rigidity of the protein [61,62]. In particular, water molecules in the distal heme pocket of myoglobin and hemoglobin form hydrogen-bonding interactions with the distal histidine and strongly affect the thermodynamics and kinetics of ligand binding to the heme iron [63,64,65]. The UV-vis spectroscopic investigation here shows that the osmolytes do not significantly perturb myoglobin and heme pocket structures. All the minor changes in UV-vis spectra could thus be explained by noting that the osmolytes weakly influence the structure of water around the protein and inside the heme pocket, as well as the structure of the heme. After the introduction of the CO into the heme pocket, the structure of the heme pocket becomes more rigid because of the hydrogen bonding interactions between the CO and distal histidine. 

### 2.2. FTIR Spectroscopy

**Figure 2:**
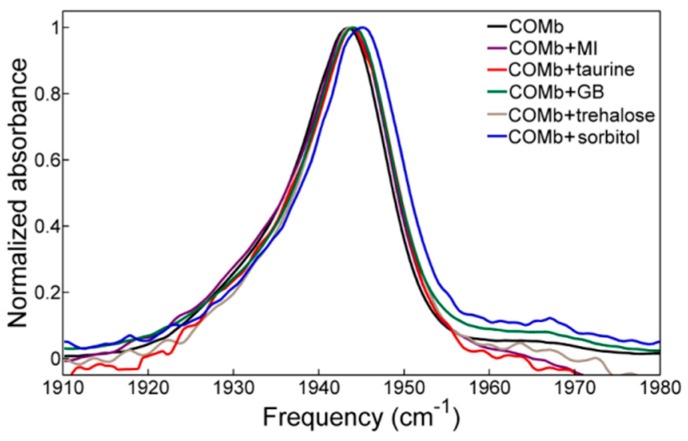
FTIR spectra of carbonmonoxy-myoglobin in various osmolytes solutions. “MI” stands for myo-inositol, and “GB” for glycine betaine. The concentrations of the osmolytes are 3.0 M for GB and sorbitol, 1.5 M for trehalose and 0.9 M for MI and taurine.

The FTIR spectra of COMb in different osmolyte solutions are plotted in Figure 2, and their fitting results are summarized in Table 1. In each of the FTIR spectra, a strong singlet peak appears, but its lineshape is highly asymmetric, so that it can be fitted with two Voigt functions and an additional shoulder band at around 1965 cm^−1^. It is known that these structures originate from different COMb conformers, more precisely, His64 N_ε_-H or N_δ_-H tautomers, which are believed to differ in the relative distance and angle of His64 with respect to the ligand, CO [31,39]. The IR peaks at 1933 cm^−1^, 1944 cm^−1^, and 1965 cm^−1^ are assigned to the A_3_, A_1_ and A_0_ conformers, respectively.

Unlike the UV-vis spectra, the CO stretch FTIR band changes upon the addition of osmolytes, which clearly indicates that these influence the vibrational solvatochromic environment around the CO probe. In the presence of the osmolytes in the COMb solutions, the strongest A_1_ peak becomes blue-shifted in the order: myo-inositol and taurine < glycine betaine < trehalose < sorbitol. Interpretation of the results needs to be done with care because of the fitting uncertainty, but the blueshift of the peak for the samples with the addition of the osmolytes in comparison to pure COMb is always reproducible. The blueshift of the CO stretch IR peak indicates that these osmolyte molecules cause a weakening of the hydrogen-bonding interaction with CO or of the d-π back bonding between CO and Fe in the heme. The magnitude of frequency shift in the sorbitol solution is as large as ~1.5 cm^−1^. Based on the observation that the CO stretching frequency shifts but the UV-vis spectrum of heme in COMb does not, we suggest that the addition of osmolytes changes the electrostatics around CO without changing the structure of the active site. In addition, the conservation of the spectral lineshape (Figure 2) for all the osmolyte solution samples considered here indicates that they do not cause any significant changes in the distribution of local conformations around the CO ligand binding to the heme. 

### 2.3. Polarization-Controlled IR Pump-Probe Spectroscopy

**Figure 3:**
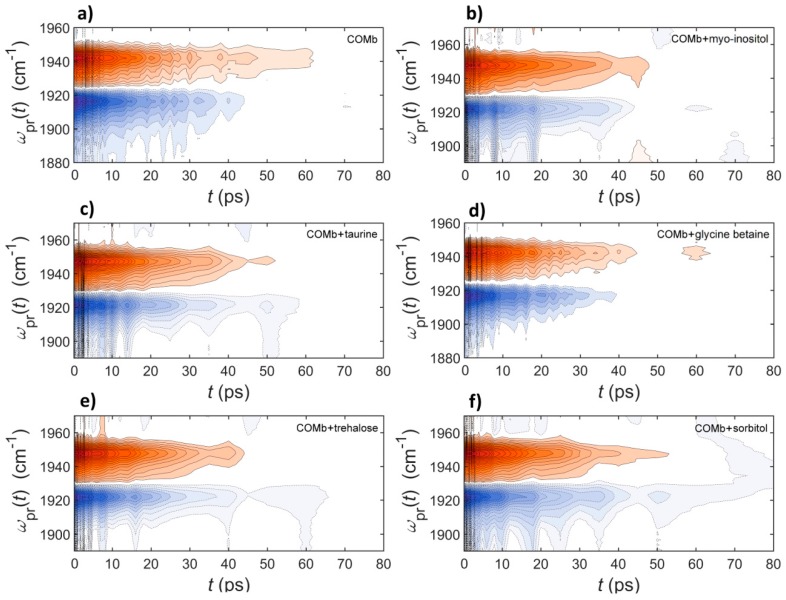
Isotropic pump-probe spectra of carbonmonoxy-myoglobin in the following solutions: (**a**) phosphate buffer in D_2_O, (**b**) 0.9 M myo-inositol, (**c**) 0.9 M taurine, (**d**) 3.0 M glycine betaine, (**e**) 1.5 M trehalose and (**f**) 3.0 M sorbitol.

**Figure 4:**
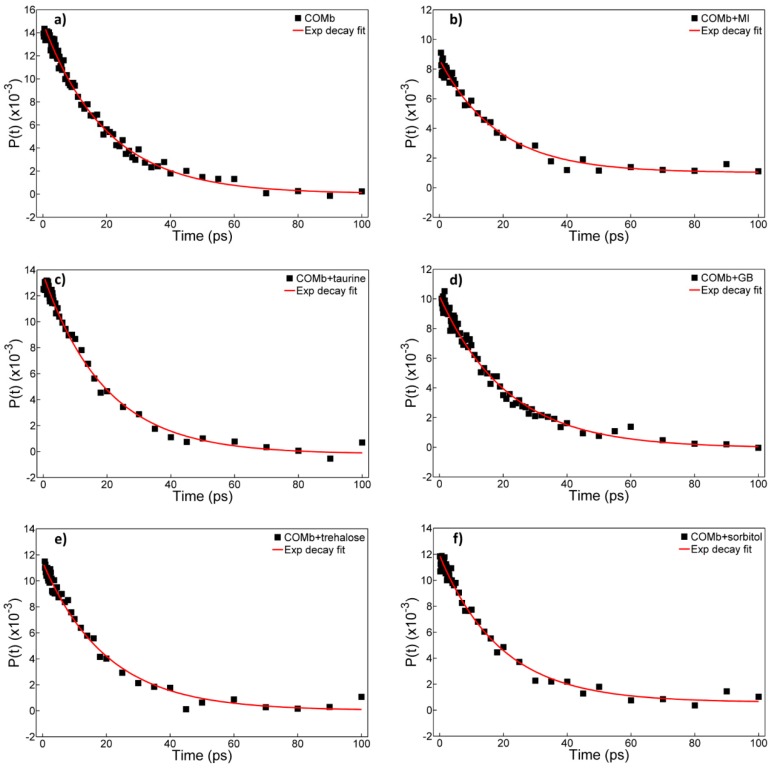
Slices of the pump-probe spectra of COMb in different osmolytes solutions for chosen frequencies of the positive peak and their single exponential decay fitting functions. “GB” stands for glycine-betaine, and “MI” for myo-inositol. (**a**) phosphate buffer in D_2_O, (**b**) 0.9 M myo-inositol, (**c**) 0.9 M taurine, (**d**) 3.0 M glycine betaine, (**e**) 1.5 M trehalose and (**f**) 3.0 M sorbitol.

We next carried out time-resolved IR pump-probe experiments to further examine how the dissolved osmolytes influence the vibrational lifetime of the CO stretch mode. The isotropic pump-probe spectra for COMb without osmolytes and with five different osmolytes are shown in the Figure 3. The IR pump-probe spectra were analyzed as follows. The IR pump-probe time profiles at various probe frequencies covering both the negative (1918–1926 cm^−1^) and positive peaks (1934–1951 cm^−1^) were fitted with a single exponential decay function. Then, we considered the average decay time constants. The representative fitting results, where the decay signals at the peak of the positive (ground-state bleach and stimulated emission contributions) IR PP spectrum are used, are shown in Figure 4. The resulting lifetimes are summarized in Table 1. 

The vibrational lifetime of the CO stretch mode of COMb in an aqueous solution is found to be 19.1 ± 0.5 ps, which is in good agreement with previous reports [31,35,39]. The addition of myo-inositol to the protein solution does not cause any change in the vibrational energy relaxation, where the vibrational lifetime of the CO stretch mode is 18.9 ± 0.9 ps. However, in the cases of the other four osmolytes, we found that the CO stretch vibrational lifetime becomes longer. For instance, the vibrational lifetime for COMb in 0.9 M taurine solution is 19.7 ± 0.8 ps, in trehalose 1.5 M solution 20.5 ± 0.5 ps, in glycine betaine 3.0 M solution 20.1 ± 0.6 ps, and in sorbitol 3.0 M solution 20.4 ± 0.6 ps. Although the differences in measured lifetimes as compared to that for COMb in water are small, and they need to be treated with caution considering their uncertainty, they are reproducible experimentally. 

The measured vibrational lifetime increases in the order: COMb and COMb + MI < COMb + taurine < COMb + GB < COMb + sorbitol and COMb + trehalose. This shows that the multiple hydroxyl groups on sorbitol and trehalose have a strong influence on the vibrational dynamics of the CO stretch mode in COMb. This is consistent with the observation that sorbitol induces the largest frequency shift of the CO stretch IR spectrum. However, it is still quite puzzling that trehalose increases the vibrational lifetime equally strongly at a concentration just half of that of the sorbitol solution. 

The fact that sorbitol and trehalose induce a notable change in the vibrational lifetime is important, as it provides a clue about osmolyte effects on protein structure and function in this particular case. Taurine and glycine betaine have similar chemical structures. They have both amine and acid groups, where taurine has a sulfonic acid group. Glycine betaine is a zwitterion at neutral pH. Thus, taurine and glycine betaine are capable of modulating water solvation structure around the protein. In contrast, trehalose (a disaccharide) and sorbitol (a sugar alcohol) are larger in size than taurine and glycine, and have multiple hydroxyl groups that can form extended H-bonding networks between osmolytes as well as between osmolyte and water molecules. Myo-inositol with six OH groups on the cyclohexyl backbone carbon atoms would also have a similar capability to form large-scale aggregates via multiple intermolecular H-bonding interactions, but its low solubility could explain the absence of any notable effect on the vibrational lifetime of the CO stretch mode.

### 2.4. Circular Dichroism Spectroscopy

**Figure 5:**
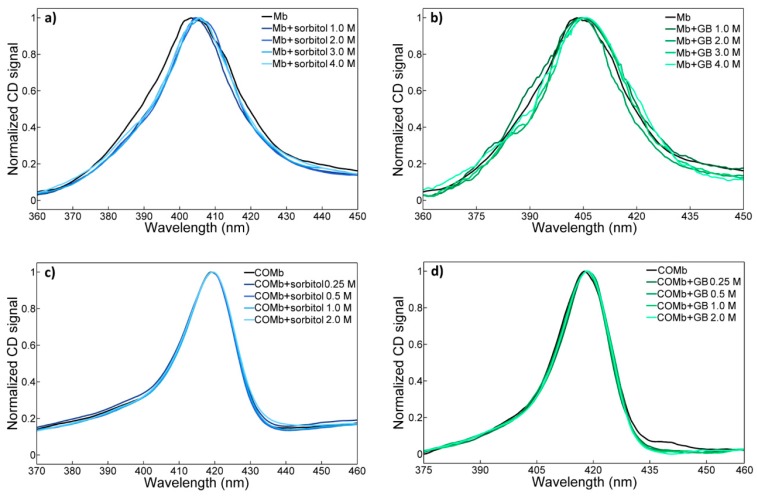
Concentration-dependent CD spectra (Soret band) of (**a**) metmyoglobin in sorbitol solutions, (**b**) metmyoglobin in glycine betaine solutions, (**c**) carbonmonoxy-myoglobin in sorbitol solutions and (**d**) carbonmonoxy-myoglobin in glycine betaine solutions.

Electronic circular dichroism (CD) is one of the most decisive spectroscopic tools that can be used to investigate structural changes of proteins induced by the addition of denaturing molecules. Here, two different CD bands are mainly discussed. The first is the Soret CD spectra of Mb and COMb in water and those in osmolyte solutions. The CD spectra of Mb and COMb in sorbitol and glycine betaine solutions are shown in Figure 5, and those for the other three osmolytes (trehalose, taurine and myo-inositol) are plotted in Figure S4. The sorbitol induces a redshift and a narrowing of the heme peak. However, we cannot find any trend in the CD spectrum, as the concentration of glycine betaine increases (note that the CD spectra of Mb solutions are particularly noisy because of the difficulty in subtracting out the background signal). Interestingly, in all the COMb solutions with or without osmolytes, the corresponding CD spectra are not affected by the presence of osmolyte in the protein solution, which is consistent with the UV-vis data. 

**Figure 6:**
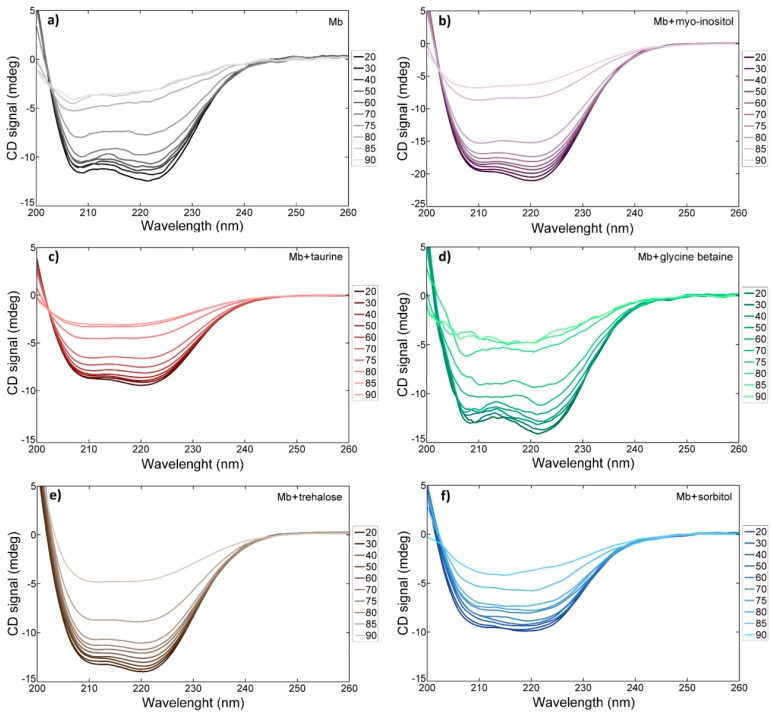
Temperature-dependent CD spectra of metmyoglobin backbone in (**a**) 7.0 pH phosphate buffer, (**b**) 0.9 M myo-inositol, (**c**) 0.9 M taurine, (**d**) 2.0 M glycine betaine, (**e**) 1.5 M trehalose and (**f**) 2.0 M sorbitol.

To investigate the osmolyte effects on the temperature-induced protein unfolding process, we measured the UV CD spectra of electronic transitions in the protein backbone. The temperature-dependent CD spectra in various solutions (phosphate buffer, 2.0 M sorbitol, 2.0 M glycine betaine, 1.5 M trehalose, 0.9 M taurine and 0.9 M myo-inositol) are shown in Figure 6. The concentrations of the studied taurine and myo-inositol solutions are comparable to the concentrations of the osmolytes in mammalian blood [45]. Concentrations of the three other osmolytes are higher than those in mammalian blood or kidney cells [44], but the osmolyte concentrations can differ by the type of cell and the conditions, and can be much higher in plants and fungus. Concentration of osmolytes in plant cells during draught can increase drastically and reach the values similar to those used in this experiment [66]. One well-known osmolyte effect is the ability to protect live cells from detrimental perturbations including thermal stress. As can be seen in Figure 6, in the cases of sorbitol and trehalose, the protein backbone CD signals show that the protein is not fully unfolded even at 90 °C, because there appears to be no notably large jump-like change in the CD spectra with increasing temperature.

**Figure 7:**
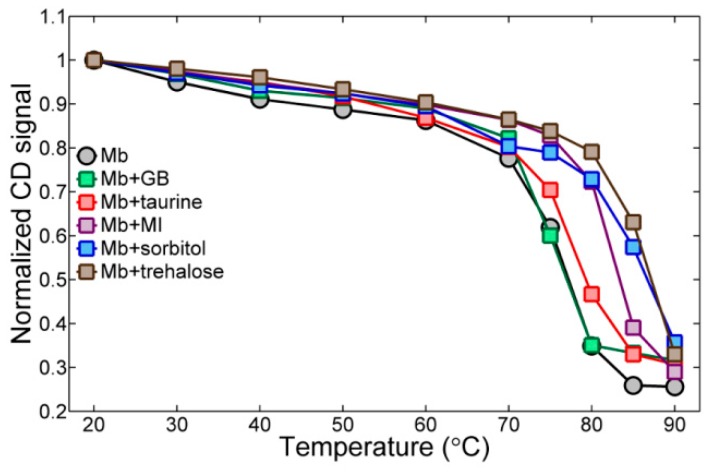
Temperature-dependent CD signal of metmyoglobin backbone in different solutions: 7.0 pH phosphate buffer, 2.0 M sorbitol, 2.0 M glycine betaine, 1.5 M trehalose, 0.9 M taurine and 0.9 M myo-inositol. In the legend, “GB” stands for glycine betaine, and “MI” for myo-inositol. The data was normalized by dividing by the minimum CD signal value. Data shown is taken at the 222 nm wavelength, which is the minimum of the backbone signal.

To estimate the unfolding transition temperature, the CD intensity at 222 nm is plotted with respect to temperature (Figure 7). By analyzing the data points in Figure 7, the unfolding of the metmyoglobin in the pH = 7.0 phosphate buffer solution occurs at around 75 °C, which is in agreement with the literature [67]. Glycine betaine, even at a high concentration of 2.0 M, does not change the unfolding temperature, which leads us to conclude that glycine betaine is a relatively weak protectant against heat. Nonetheless, osmolytes shift the unfolding temperature toward a higher temperature. In the 0.9 M taurine solution, its thermal protection effect is intermediate among the osmolytes considered here. The 0.9 M myo-inositol increases the unfolding temperature by about 8 °C (up to 83 °C) in comparison to that of metmyoglobin in a buffer solution. Among the osmolytes, sorbitol and trehalose elevate the unfolding temperature by around 15 °C.

In summary, we find that the unfolding temperature increases gradually according to the following order: Mb and Mb + GB < Mb + taurine < Mb + MI < Mb + sorbitol and Mb + trehalose. This trend is in agreement with that found in the change of CO stretch vibrational lifetime induced by osmolytes. Therefore, it is concluded that all the osmolytes influence the solvation environment around each protein to a certain degree. However, in terms of protein stability and thermal protection, notably different effects appear to be induced by each osmolyte, which are related to their characteristic chemical structures. Two small, charged molecules, glycine betaine and taurine, even at high concentrations (2.0 M), have little impact on the protein. On the other hand, myo-inositol, an osmolyte which does not cause any significant changes in the FTIR and IR pump-probe spectra, is capable of inducing a large shift in the unfolding temperature, i.e., by around 7 °C, even at a relatively low concentration of 0.9 M. The strongest effects on the protein are induced by sorbitol and trehalose. Interestingly, myo-inositol, sorbitol, and trehalose all are polyols with multiple H-bond donating and accepting sites. Consequently, they can form an extended H-bonding network structure via making multiple H-bonds with one another. Thus, it would be extremely interesting to study a possible interplay of osmolyte aggregate formation in aqueous protein-osmolyte solutions with water structure on the surface of a protein by means of a MD simulation in the future. 

## 3. Materials and Methods

### 3.1. Materials

Myoglobin from the equine heart, D_2_O, monosodium phosphate, disodium phosphate, sodium dithionite, sorbitol, glycine betaine, trehalose, taurine and myo-inositol were purchased from Sigma-Aldrich (Seoul, South Korea) and used as received. Carbonmonoxy myoglobin (COMb) was prepared by reducing metmyoglobin with sodium dithionite. Then, the COMb solution was purged with CO gas for an hour. All of the myoglobin samples for CD and UV-vis measurements were prepared using 7.0 pH phosphate buffer. COMb samples were prepared using homemade 7.0 pH phosphate buffer in D_2_O.

### 3.2. UV-vis Spectroscopy

UV-vis spectra were recorded using a Lambda 465 UV/VIS spectrometer (PerkinElmer, Seoul, South Korea) at 22 °C with a frequency resolution of 1 nm, and a quartz cell with a pathlength of 1 mm. The concentrations of myoglobin and COMb solutions were 25 and 15 μM, respectively.

### 3.3. FTIR and IR Pump−Probe Spectroscopy

FTIR spectra were recorded utilizing a VERTEX 70 spectrometer (Bruker, Ettlingen, Germany). FTIR data was collected with a frequency resolution of 1 cm^−1^ at 22 °C. For FTIR and IR pump−probe experiments, samples at a concentration of 15 mM were placed in a home-built IR cell with two 3 mm thick CaF_2_ windows and a 56 µm thick Teflon spacer.

Experimental details about our femtosecond mid-IR laser setup have been described elsewhere [68]. IR pump-probe experiments were performed at the Seoul Center belonging to the Korea Basic Science Institute (KBSI). In short, a 800 nm pulse is generated from the femtosecond Ti:Sapphire oscillator (Tsunami, Spectra-Physics, Santa Clara, California, USA) and regenerative amplifier (Spitfire, Spectra-Physics, Santa Clara, California, USA). These pulses are used to pump an optical parametric amplifier (OPA) to produce near IR pulses centered at ~1.4 μm and ~1.9 μm. These near IR pulses are focused onto a 2.0 mm thick AgGaS_2_ crystal where the difference frequency generation process occurs, resulting in the generation of mid-IR pulses. These mid-IR pulses are split by a ZnSe beam splitter into pump and time-delayed probe pulses with an intensity ratio of 9:1, and the time delay between those pulses is controlled by a motorized linear stage placed on the probe beam path. Pump and probe pulses are focused onto a sample and the generated signal is frequency-resolved with a monochromator and detected by a 64-element MCT array detector at each time delay. As the time delay increases, the intensity of the pump-probe signal decays due to both vibrational and orientational relaxation processes. To separately measure the vibrational population relaxation (*P*(t), isotropic signal) and the rotational relaxation (*r*(t), anisotropic signal) contributions to the pump-probe signal, the relative polarization of the probe beam with respect to that of the pump was controlled using a rotating polarizer, and two polarization conditions were considered, i.e., parallel *S*_‖_(t) and perpendicular *S*_⊥_(t) signals. The isotropic signal responsible for population relaxation was obtained from the parallel and perpendicular signals, using the relation:

(1) $$P\left(t\right)=\frac{S_{\|}\left(t\right)+2S_{⊥}\left(t\right)}{3}$$

### 3.4. Circular Dichroism Spectroscopy

Circular dichroism (CD) spectra were recorded using a J-815 CD spectrometer (Jasco, Tokyo, Japan) with a PTC-423S/15 temperature controller (Jasco, Tokyo, Japan). All the measurements were conducted under nitrogen gas of 1.0 MPa. The CD spectra were measured at 22 °C with a frequency resolution of 0.2 nm. The concentrations of Mb and COMb solutions were 25 μM and 15 μM, respectively. For the Soret and Q band CD spectra measurements, a quartz cell with the pathlength of 10 mm was used. For of the temperature-controlled CD spectra of the protein backbone, the pathlength used was 0.2 mm. During the temperature-controlled data measurement, the temperature was raised by 10 °C in ~10 min, and then the sample was left at the target temperature for 10 min to equilibrate. A real temperature of the solution in the CD cell was determined with a thermocouple detector in each measurement.

## 4. Conclusions

In the present work, we have studied the effects of five different osmolytes (sorbitol, trehalose, myo-inositol, glycine betaine and taurine) on the structure and dynamics of myoglobin, using a variety of spectroscopic methods, e.g., UV-vis, CD, FTIR and IR pump-probe spectroscopy. 

The UV-vis spectroscopic results show that the addition of the osmolytes does not cause any strong perturbation to the inner active site of the protein, and as a consequence, there is no structural change to the core part of the protein. The observed changes in the UV-vis spectra of carbonmonoxy myoglobin are smaller than those of for metmyoglobin, probably because the insertion of the CO into the heme pocket causes the structure of the pocket to become less penetrable to solvent molecules due to the hydrogen bonding interactions between the CO and distal histidine. The same trend was observed in the corresponding CD spectra. 

The changes in the CO stretch FTIR spectra of COMb upon the addition of osmolytes are again small, but a notable blue-shift of the peak frequency of the CO band was observed when osmolytes were added to the solution, when compared with the protein buffer solution. Sorbitol induces the largest frequency shift. Interestingly, the change in the CO vibrational lifetime is in good correlation with the frequency shift of the FTIR spectrum. More specifically, the measured vibrational lifetime increases gradually in the order: COMb (19.1 ± 0.5 ps) and COMb + MI (18.9 ± 0.9 ps) < COMb + taurine (19.7 ± 0.8 ps) < COMb + GB (20.1 ± 0.6 ps) < COMb + sorbitol (20.4 ± 0.6 ps) and COMb + trehalose (20.5 ± 0.5 ps). This indicates that these osmolytes, except for myo-inositol, induce changes in the FTIR spectrum and the vibrational lifetime of COMb, because they influence the solvation environment around the CO probe. The temperature-dependent CD spectra show that the myoglobin unfolding temperature increases in the following order: Mb and Mb + GB (75 °C) < Mb + taurine (79 °C) < Mb + MI (83 °C) < Mb + sorbitol and Mb + trehalose (90 °C). This trend is very similar to that of the CO stretch vibrational lifetime. Additionally, it can be directly connected to the osmolytes structures and their hydrogen-bonding abilities. This result is also in agreement with the vitrification hypothesis, and supports even further the proposition that such effects may be a property of all sugars and polyols [58].

In summary, our experimental results do not provide any decisive evidence for the direct interaction of the osmolytes with protein. Rather, the observed spectroscopic changes are consistent with the notion that the osmolyte molecules influence the structure of water around the protein and inside the heme pocket, which then affect protein structure indirectly. Both the blueshift of the CO stretch IR spectrum and the slow-down of CO vibrational energy relaxation upon the addition of osmolytes are indications of changes in the solvatochromic environment around the CO. This observation is in good correlation with the protein melting temperature, where small osmolytes like taurine and glycine betaine do not strongly change the protein solvation environment, but polyols like trehalose and sorbitol having multiple H-bonding sites are found to be protein-protectants.

## Figures and Tables

**Table 1:** Spectral properties of COMb in different osmolytes solutions. “MI” stands for myo-inositol, and “GB” for glycine betaine.

		COMb	COMb + MI 0.9 M	COMb + Taurine 0.9 M	COMb + GB 3 M	COMb + Trehalose 1.5 M	COMb + Sorbitol 3 M
ω_center_ (cm^−1^)	A_1_	1943.0	1943.8	1943.8	1943.9	1944.0	1944.6
	A_3_	1930.5	1931.3	1933.2	1935.6	1935.2	1933.9
	A_0_	1967.6	1963.2	1967.3	1967.2	1966.9	1965.1
T_1_ (ps)		19.1 ± 0.5	18.9 ± 0.9	19.7 ± 0.8	20.1 ± 0.6	20.5 ± 0.5	20.4 ± 0.6

## Supplementary Materials

The following materials are available online. Figure S1: UV-vis spectra of myoglobin (Mb) and carbonmonoxy myoglobin (COMb); Figure S2: Chemical structures of the used osmolytes; Figure S3: Concentration dependent UV-VIS spectra of: (**a**) Mb in trehalose solutions, (**b**) COMb in trehalose solutions, (**c**) Mb in taurine solutions, (**d**) COMb in taurine solutions, (**e**) Mb in myo-inositol solutions and (**f**) COMb in myo-inositol solutions; Figure S4: Concentration dependent CD spectra of: (**a**) Mb in trehalose solutions, (**b**) COMb in trehalose solutions, (**c**) Mb in taurine solutions, (**d**) COMb in taurine solutions, (**e**) Mb in myo-inositol solutions and (**f**) COMb in myo-inositol solutions; Figure S5: The near-UV region CD spectra of metmyoglobin in various osmolytes solutions.
