# Supplementary material for: Do Osmolytes Impact the Structure and Dynamics of Myoglobin?
Source: Molecules. 2018 Dec 3;23(12):3189. doi: 10.3390/molecules23123189 (PMC6321238; doi:10.3390/molecules23123189)
Supplement: Supplementary file 1 [file molecules-23-03189-s001.pdf]

Supporting Information for

Do the Osmolytes Impact the Structure and Dynamics of Myoglobin?

Dorota Kossowska [1], Kyungwon Kwak [1,2,*] and Minhaeng Cho [1,*]

[1] Center for Molecular Spectroscopy and Dynamics, Institute for Basic Science (IBS), Seoul 02841, Republic of Korea;
[2] Department of Chemistry, Korea University, Seoul 136-713, Korea;
* Correspondence: kkwak@korea.ac.kr (K.K.), mcho@korea.ac.kr (M.C.);

* Author to whom correspondence should be addressed: kkwak@korea.ac.kr (K.K.), mcho@korea.ac.kr (M.C.).

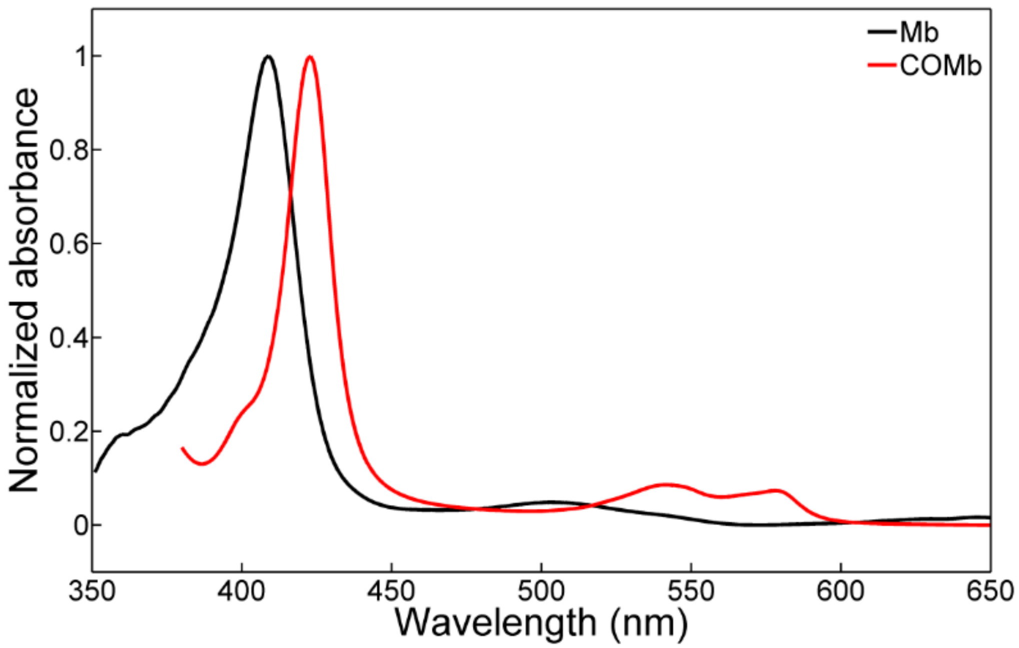

Figure S1. UV-VIS spectra of myoglobin (Mb) and carbonmonoxy myoglobin (COMb).

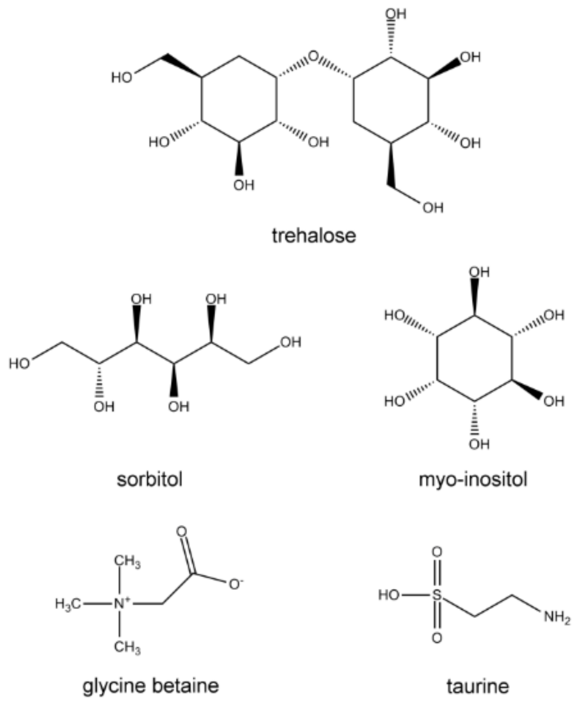

Figure S2. Chemical structures of the used osmolytes.

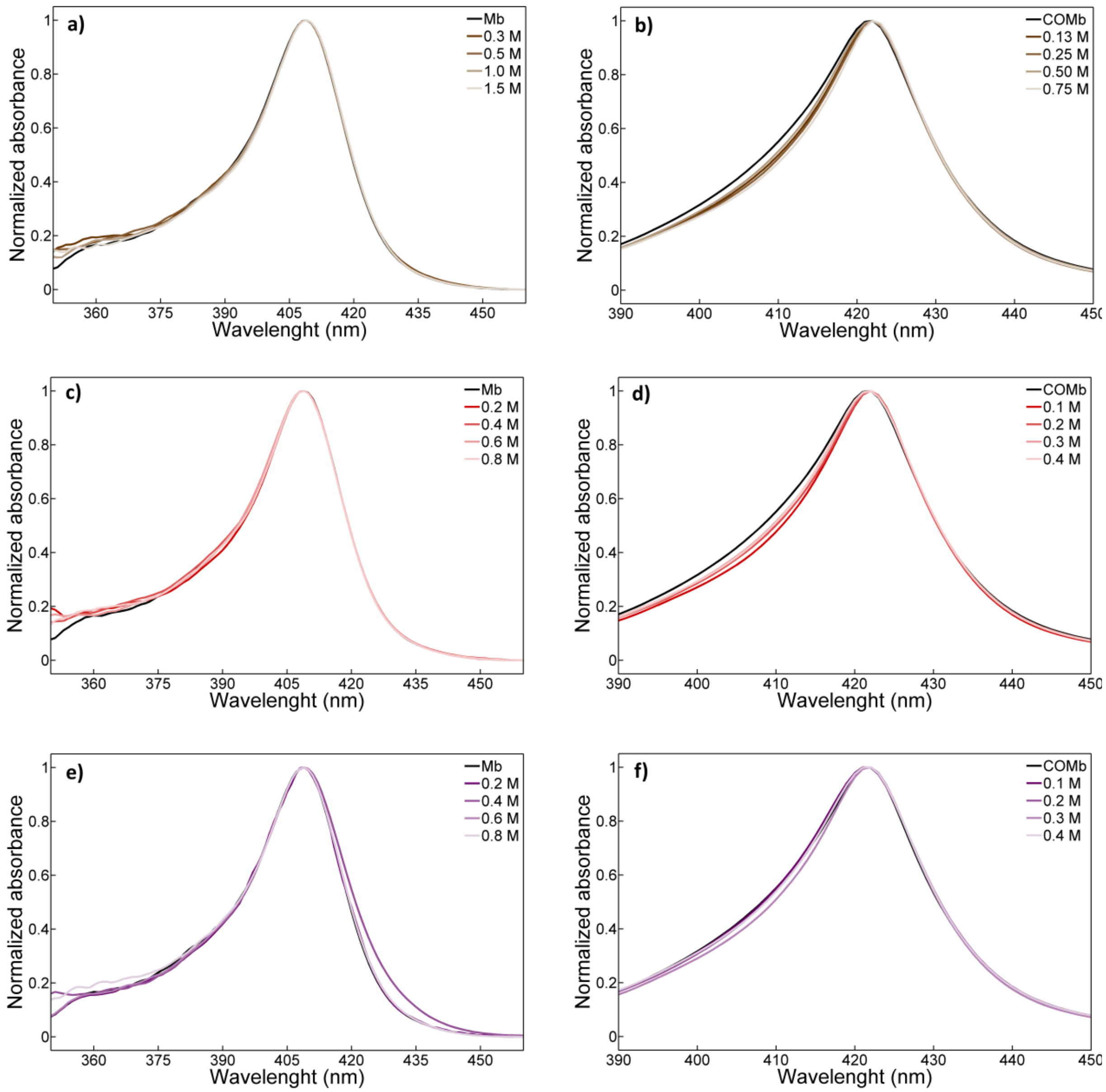

Figure S3. Concentration dependent UV-VIS spectra of: (**a**) Mb in trehalose solutions, (**b**) COMb in trehalose solutions, (**c**) Mb in taurine solutions, (**d**) COMb in taurine solutions, (**e**) Mb in myo-inositol solutions and (**f**) COMb in myo-inositol solutions.

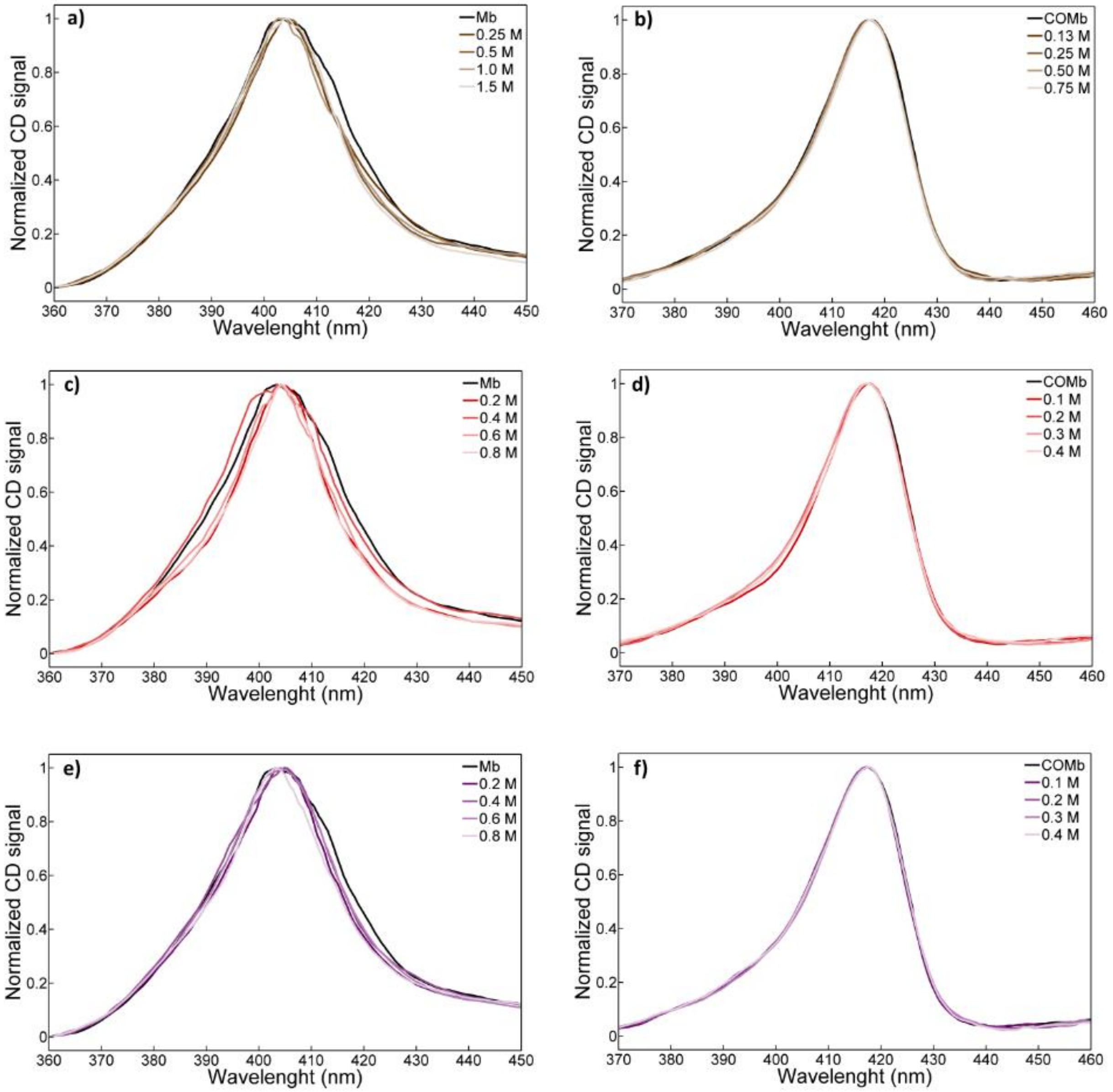

Figure S4. Concentration dependent CD spectra of: (**a**) Mb in trehalose solutions, (**b**) COMb in trehalose solutions, (**c**) Mb in taurine solutions, (**d**) COMb in taurine solutions, (**e**) Mb in myo-inositol solutions and (**f**) COMb in myo-inositol solutions.

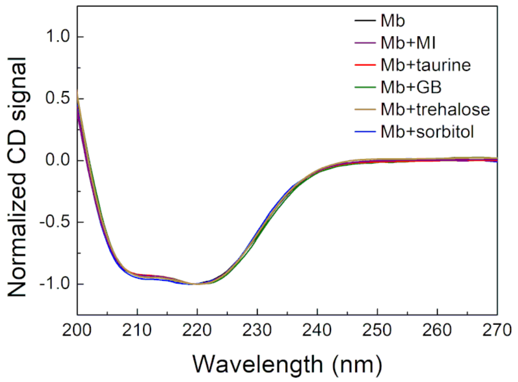

Figure S5. The near-UV region CD spectra of metmyoglobin in various osmolytes solutions. The MI stands for myo-inositol and GB for glycine betaine. The concentrations of the osmolytes are 3.0 M for GB and sorbitol, 1.5 M for trehalose and 0.9 M for MI and taurine.

Unfortunately, the changes induced by the dissolved osmolytes in the near-UV region CD spectra of metmyoglobin, which reports the protein backbone structure, are also marginal and it is difficult to observe any dependence of the spectrum on the type of the osmolyte.
